# Can a few non‐coding mutations make a human brain?

**DOI:** 10.1002/bies.201500049

**Published:** 2015-08-25

**Authors:** Lucía F. Franchini, Katherine S. Pollard

**Affiliations:** ^1^ Instituto de Investigaciones en Ingeniería Genética y Biología Molecular (INGEBI)Consejo Nacional de Investigaciones Científicas y Técnicas (CONICET)Buenos AiresArgentina; ^2^ Gladstone InstitutesSan FranciscoCAUSA; ^3^ Institute for Human Genetics, Department of Epidemiology & BiostatisticsUniversity of CaliforniaSan FranciscoCAUSA

**Keywords:** development, evolution, gene regulation, neuroscience

## Abstract

The recent finding that the human version of a neurodevelopmental enhancer of the *Wnt* receptor *Frizzled 8* (*FZD8*) gene alters neural progenitor cell cycle timing and brain size is a step forward to understanding human brain evolution. The human brain is distinctive in terms of its cognitive abilities as well as its susceptibility to neurological disease. Identifying which of the millions of genomic changes that occurred during human evolution led to these and other uniquely human traits is extremely challenging. Recent studies have demonstrated that many of the fastest evolving regions of the human genome function as gene regulatory enhancers during embryonic development and that the human‐specific mutations in them might alter expression patterns. However, elucidating molecular and cellular effects of sequence or expression pattern changes is a major obstacle to discovering the genetic bases of the evolution of our species. There is much work to do before human‐specific genetic and genomic changes are linked to complex human traits.

Also watch the Video Abstract.

**Abbreviation**
HAR: human accelerated region

## Introduction

As the human and chimpanzee lineages split, both species have acquired many distinct behaviors, morphological characteristics, and molecular phenotypes 1, 2. Some of the most salient human‐specific traits reside in our brain or involve our unique cognitive abilities 3, 4, 5. Although numerous differences between humans and other primates have been described (2; http://carta.anthropogeny.org/content/about-moca), identifying the DNA alterations responsible for these evolutionary changes is a much more difficult task. This is not because we lack candidates: many human‐specific DNA sequences have been identified. These discoveries began prior to genome sequencing. The first differences between the human and chimp genomes were discovered using chromatin‐stained banding techniques that allowed identification of the fusion of two ancestral ape chromosomes to form human chromosome 2, human‐specific constitutive heterochromatin C bands on chromosomes 1, 9, 16 and Y, and human‐specific pericentric inversions on chromosomes 1 and 18 6. It is now known that many of these structural variants have altered gene expression or downstream phenotypes in humans. For example, the pericentric inversion of chromosome 1 contains copy number increases of developmental genes, and it has been associated with human developmental and neurogenetic diseases 7, 8, 9, 10, 11. After the sequencing of the human genome 12, 13 and many other mammalian genomes, particularly the chimpanzee and the macaque 14, 15, we now have several detailed genome‐wide catalogs of human‐specific genome changes that include chromosome segmental duplications resulting in the appearance of new human genes, differences in splicing, genes that underwent positive selection in humans, and evolutionarily conserved non‐coding sequences with many human‐specific mutations (reviewed in 7, 16, 17, 18). The challenge we now face is how to link specific genetic differences to uniquely human traits.

A number of factors make this a difficult task. First, we have millions of genetic candidates to sort through, and many of these are likely to be dead ends. The neutral theory of molecular evolution, coupled with redundancy in biological networks, suggests that many human‐specific DNA changes had little effect on our biology. On the other hand, most uniquely human traits are complex, and there is no doubt that they are encoded by a combination of mutations in different genomic loci. To make matters worse, hypotheses about causal relationships between human genotypes and phenotypes are difficult to test given the obvious limitations on experimentation and genetic manipulation in humans and non‐human primates. This challenge is partially addressed by engineering human or primate DNA into model organisms, such as mice or fish, and comparing molecular and organismal phenotypes. But the interpretation of human genetic changes in model organisms is challenging, and many hurdles remain 19. Finally, we still have much to learn about the development of all organs, and in particular the complicated architecture of the brain, including how form leads to function. Without this knowledge, it is extremely difficult to predict which mutations have altered our biology during human evolution.

In this context, we carefully analyze a recently published study by Boyd and co‐workers 20, which sheds light on the consequences of human‐specific mutations in a non‐coding region located upstream of the *Wnt* receptor *Frizzled 8* (*FZD8*). The authors characterize the region as a transcriptional enhancer of *FZD8* and then attempt to link its accelerated evolution in humans to changes in neurodevelopment, including spatial and temporal differences in neuronal gene expression leading to a faster cell cycle of neural progenitor cells. The significance of these discoveries is evaluated along side other recent work aiming to functionally characterize uniquely human DNA sequences and elucidate their contributions to the evolution of human traits.

## The human brain: What is different about it?

The human brain has all the basic characteristics of a typical mammalian brain such as the six‐layered cortex or neocortex. In addition, our brain also has the typical features of a primate brain: an unusually large neocortex, a big visual cortex and a lateral prefrontal cortex 21. Despite these overall similarities, the human brain has several features that make it unique. The anatomy and development of the human brain diverged in several key ways from those of other primates during evolution. Humans have the largest number of neurons of any primate: approximately 86 billion 22 compared with an estimated 28 and 33 billion neurons in chimpanzee and gorilla brains, respectively 23. On the other hand, our brain is not the largest on earth, being outranked by brains of cetaceans and elephants 24. However, although the elephant brain has about 251 billion neurons, only 5.6 billion (2.2%) are cortical, the majority being concentrated in the cerebellum (97%; 25). In contrast, 20.9% of all neurons in the human brain are cortical, which is more than 10% greater than the cortical proportion in any other mammal 26. Thus, the human cortex is proportionally the largest (84% of the entire brain mass), and it contains the most neurons (85 billion) of any mammal 23, 25, 26, although it is debated whether our neocortex is particularly unique 27, 28.

Beyond numbers, the human brain appears also to be unique in its organization. Non‐invasive brain imaging techniques, such as diffusion‐tensor imaging, made it possible to study long‐range interactions of the cortex and revealed differences in cortical connections in human brains compared with those of chimpanzees and macaques 29. In addition, post‐mortem studies showed that the human brain is also unique in terms of cellular and histological organization of the cortex 30, 31, 32.

To understand the evolution of our species' higher order cognitive abilities, including abstract thinking, long term planning, and an extraordinary ability to produce and elaborate a complex language, we must answer two challenging questions. The first is how to link human cognition to number of neurons, brain size, a highly developed cortex, and particular neuroanatomical differences. The neurobiological bases of our linguistic capacity, for example, are not completely understood, because the main areas controlling language in the brain are also present in chimpanzees 33, 34. The second challenge is to connect DNA changes to uniquely human neurobiology. Although some advances have been made towards understanding the genetics underlying human cognitive traits, such as our spoken language 17, 18, very little is known about the anatomical and molecular mechanisms through which these genetic differences are expressed in the human brain. This is the question addressed by Boyd and colleagues.

## An accelerated non‐coding sequence may have altered brain development in human evolution

Boyd et al. designed a study to identify genome sequences involved in the evolution of the unique features of the human cortex. Specifically, they focused on uniquely human gene regulatory enhancers active during neurodevelopment. The authors took advantage of previously identified catalogs of nearly 2,700 human accelerated regions (HARs), which are non‐coding sequences that changed significantly in the human lineage after having been highly conserved across mammalian evolution 35, 36, 37, 38, 39. Conserved non‐coding sequences have been hypothesized to contain much of the regulatory machinery that controls the time, place and mode of expression of genes 40, 41, 42, 43, and human‐specific mutations may alter this function. To provide further evidence of regulatory function and to focus their study on the developing brain, Boyd and co‐workers crossed the list of HARs with publicly available datasets of genome regions displaying epigenetic signatures of enhancer activity in various neurodevelopmental cell types (Supplemental Table S1 in 20). Specifically, the authors used ChIP‐seq data measuring genome sequences bound by (i) the co‐activator p300 (a component of enhancer‐associated protein assemblies) in mouse forebrain tissue at embryonic day 11.5 (E11.5) 44, (ii) the key neurogenesis transcription factors Pax6 and Sox2 in mouse embryonic cortex tissue at E12.5 45 and neural stem cells 46, and (iii) histones with modifications indicative of active enhancers such as H3K4me1 or H3K27ac in neural progenitor cells 47, 48. This analysis allowed them to identify 106 non‐overlapping HARs containing transcriptional enhancer epigenetics marks. From these putative neural enhancers they selected six (HARE1‐6) that were located near genes known or predicted to be involved in corticogenesis, and they performed enhancer assays in transgenic mice. Although three of the selected HAREs displayed enhancer activity in the developing cortex of transgenic mice, the authors selected HARE5, also known as Accelerated Non‐Coding element 516 (ANC516) 35, for further studies because of its highly consistent enhancer activity and location nearby the developmental gene *FZD8*.

**Figure 1:**
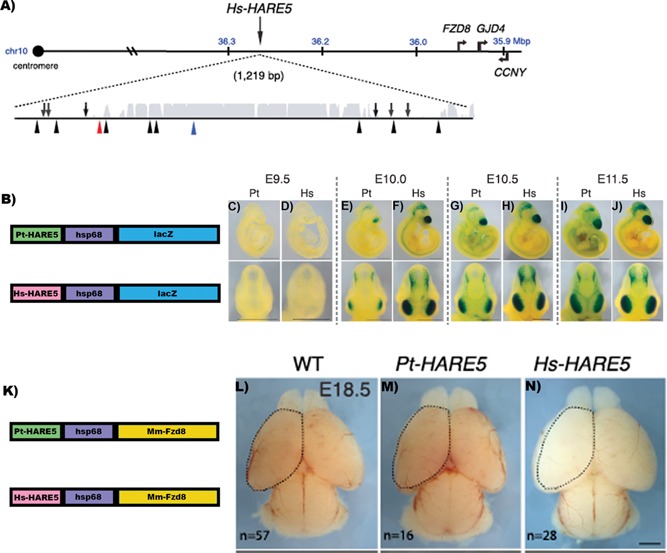
HARE5 gained expression compared with chimpanzee ortholog, driving increases in cortical size in overexpression assays. **A:** Schematic of Hs‐HARE5 locus on human chromosome 10 (hg19) showing its location relative to genes nearby including *FZD8*. The 1,219‐bp‐long HARE5 genomic locus with enhancer activity includes the original 619 bp human‐accelerated sequence and flanking sequences. Represented below is a conservation track for the HARE5 locus, showing a region of high‐sequence conservation (gray). Also shown are lineage‐specific mutations for chimpanzee (six; arrows, above line) and human (ten; arrowheads, below line), including one Denisovan (red) and one known human polymorphism (blue). **B:** Schematic of Pt‐HARE5::lacZ and Hs‐HARE5::lacZ constructs. Representative images of mouse embryos (stable transgenic lines) showing activity (blue LacZ staining) of Pt‐HARE5::LacZ (**C**, **E**, **G**, and **I**) and Hs‐HARE5::LacZ (**D**, **F**, **H**, and **J**). **K:** Schematic of Pt‐HARE5::Fzd8 and Hs‐HARE5::Fzd8 constructs. **L** to **N:** Whole‐mount E18.5 mouse brains from the indicated genotypes (n, number of brains examined) showing differences in cortical size. A dotted line was drawn on the WT cortex in (**L**) to indicate dorsal cortical area and was then superimposed on transgenic cortices in (**M**) and (**N**). Modified with permission from 19.

To link the evolution of HARE5 to human neocortical development, Boyd et al. first generated transgenic mice carrying a reporter gene under the control of the human HARE5 enhancer (Hs‐HAR5::LacZ). These mice expressed B‐galactosidase in the lateral forebrain, dorso‐lateral midbrain, spinal cord, and retina (Fig. 1). On the other hand, transgenic mouse embryos carrying the orthologous chimpanzee HARE5 sequence (Pt‐HARE5::lacZ) also displayed B‐gal activity in the same tissues. But at E10.0 and E10.5, Pt‐HARE5 drove weaker and more limited activity in the developing cortex compared with the human version (Fig. 1). There are other genes in the HARE5 region (Fig. 1) that could be the target of this enhancer action: the ankyrin repeat domain 30A (ANKRD30A) (around 1 Mb away), a gene expressed in breast and testis; the gap junction protein, delta 4 (GJD4; located at 350 kb), and CYCLIN Y (CCNY; located at around 400 kb). Then, in order to show which gene is the target of the enhancer activity of HARE5 the authors performed chromosome conformation capture (3‐C) to test for physical association between mouse HARE5 and the core *Fzd8* promoter in E12.5 mouse neocortices. They found that mouse HARE5 interacts with the *Fzd8* promoter in the neocortex, but it does not interact with this promoter in liver of the same stage embryos, showing that HARE5 is a *Fzd8* regulatory region in the developing cortex.

In addition, the authors generated transgenic mice carrying the human or chimp HARE5 sequences driving the expression of the mouse *Fzd8* coding sequence. Then they compared several parameters of cortical development, including cortical size, cell cycle state and duration in neural progenitors at E12.5. The results revealed that overexpression of *Fzd8* driven by Hs‐HARE5 produced faster progenitor cell cycle in the developing brain and increased neocortical size compared with mice where *Fzd8* is controlled by chimpanzee HARE5 or wild‐type mice 20.

Although the findings by Boyd and co‐workers are very suggestive, the further demonstration of the role of HARE5 in human evolution will require additional studies. For example, it would be necessary to use knock‐in strategies, rather than overexpression, to better demonstrate changes in phenotypes. The location of HARE5 near the centromere makes this very challenging. From the data included in the paper, we do not know whether the same number of copies of the Hs‐HARE5‐*Fzd8* and Pt‐HARE5‐*Fzd8* transgenes were inserted in the genome of compared transgenic mice and if this could influence the differences in phenotype observed. In addition, we lack information about the locus where the transgenes where inserted, and this is another factor to take into account when analyzing transgenic mouse studies. In that sense, locus‐directed insertions of transgenes could produce cleaner results. Another issue is that HARE5 contains 10 human‐specific changes as well as 6 chimpanzee‐specific changes. To determine how the changes in each lineage affected the function of this sequence, it would be ideal to perform functional studies with computationally reconstructed ancestral sequences or sequences from other primates and mammals to definitively show that the human mutations, and not the chimpanzee mutations, altered HARE5 function. Then, it would be helpful to compare brain architecture, as well as counts and ratios of different embryonic cell types, in the context of human versus ancestral HARE5. Another issue is that because of the lack of information about the expression of *FZD8* in human and chimpanzee brains, we do not know if this gene is in fact differentially expressed in these two species. Finally, it will be important to generate adult mice carrying human HARE5 and measure brain size, neuronal cell counts, and cognitive and behavioral traits.

## Why focus on gene regulatory regions?

Before the genomic era that made it possible to identify conserved non‐coding sequences, most studies searching for the genetic bases of the evolution of human traits focused on protein‐coding regions. These projects identified several brain genes that underwent positive selection in the human genome (for review see 1, 8). In addition, other studies identified new human genes that appeared after the divergence between the human and chimpanzee lineages and linked these genes to the evolution of particular aspects of the human brain 7, 49, 50. So, one might wonder why Boyd and others have looked beyond protein‐coding genes to understand human evolution. First, there are more than twice as many DNA bases in conserved non‐coding regions compared with protein‐coding regions, making them a larger target for evolutionary innovation. Moreover, the high similarity of human and chimpanzee proteins, which has been known for many years 51, 52 and was confirmed with the sequencing of the chimpanzee genome 14, makes it very unlikely that coding changes are the primary source of phenotype differences between the two species. Supporting the potential for the relatively small number of non‐coding mutations in HARs to play a major role in human evolution, studies in other species found that lineage‐specific evolution of regulatory sequences can drive phenotypic changes (reviewed in 53, 54). These studies highlight the importance of studying non‐coding regulatory sequences to unravel the evolution of humans. Boyd and co‐workers pursued this hypothesis by leveraging HAR sequences as raw material upon which to first design studies to test if HARs function as neurodevelopmental enhancers and then attempt to find the possible impact of human mutations on this function.

## Many human accelerated non‐coding sequences function as enhancers

Boyd and co‐workers were not the first group to test HARs for gene regulatory function in the developing embryo (Table 1). The first functional study involved HAR1 38, which encodes a long noncoding RNA that is co‐expressed with reelin in Cajal–Retzius neurons in the developing human neocortex from 7 to 19 gestational weeks, a crucial period for cortical neuron specification and migration. Despite extensive sequence changes, the expression pattern of HAR1 in the developing cortex has been highly conserved since the divergence of hominoids and Old World monkeys 38, and no clear phenotypic consequences of HAR1 mutations have been identified to date. The first HAR to be tested as a developmental enhancer was HAR2/HACNS1 55. Using a transgenic mouse enhancer assay, the authors found that the human HAR2/HACNS1 sequence drove strong and reproducible expression in the developing limb bud, pharyngeal arches, ear, and eye at E11.5. In contrast, the chimpanzee and macaque ortholog failed to drive reproducible reporter gene expression in the distal limb bud, while driving expression to the base of the limb 55. Although the authors suggested that this change was produced by adaptive evolution, the pattern of substitutions is more consistent with biased gene conversion, and it has been suggested that the functional change in expression pattern may have been driven by destruction of a repressor binding site 56.

**Table 1:** HARs tested in functional assays that showed differences with the chimpanzee ortholog

HARs	Genome location (hg19)	Closest gene	Experimental approach	Biological function	Domain of expression	Reported functional difference	Postulated phenotypic association		References
HAR1	chr20:61,733,447‐61,733,630	*HAR1F*, HAR1R	qPCR; in situ hybridization	RNA gene	Cajal‐Retzius neurons	RNA secondary structure		Brain development	37
HACNS1/HAR2	chr2:236773664‐236774209	AGAP1 (CENTG2), GBX2	Transgenic mice	Enhancer	Limb, branchial archs	Gain of function		Limb development	54
2xHAR.142	chr14:34048651‐34048815	*NPAS3*	Transgenic mice	Enhancer	Forebrain, midbrain, spinal cord	Gain of function		Brain development	57
HAR202	chr14:34045570‐34045772	*NPAS3*	Transgenic zebrafish	Enhancer	Central nervous system	Loss of function		Brain development	56
ANC516	chr10:36238421‐36239039	*FZD8*	Transgenic mice	Enhancer	Forebrain, midbrain, spinal cord	Gain of function		Brain development	19
2xHAR.238	chr2:121832784‐121833140	*GLI2, TFCP2L1*	Transgenic mice	Enhancer	Forebrain, midbrain, hindbrain and spinal cord	Loss of function		Brain development	58
2xHAR.114	chr20:30425200‐30425393	*MYLK2*, *FOXS1*	Transgenic mice	Enhancer	Limb and spinal cord	Loss of function		Limb development	58
2xHAR.164	chr2:133346894‐133347089	*ANKRD30BL*, GPR39*,* LYPD1*,* NCKAP5	Transgenic mice	Enhancer	Forebrain, midbrain, hindbrain, spinal cord	Gain of function		Brain development	58
2xHAR.170	chr5:153640309‐153640363	GALNT10, SAP30L, HAND1	Transgenic mice	Enhancer	Isthmus and spinal cord	Loss and gain of function		Brain development	58
HAR25	chr4:182271922‐182272882	*ODZ3*	Transgenic mice	Enhancer	Eye	Loss of function		Eye development	58

These first functional studies were followed by several investigations that examined bigger sets of predicted HAR enhancers. Analyzing genomic loci with the largest clusters of HARs, Kamm and co‐workers 57 found that the brain transcription factor *NPAS3* is associated with 14 HARs, 11 of which act as transcriptional enhancers in a transgenic zebrafish reporter assay. They further showed that human HAR202 lost developmental enhancer capacity compared with the orthologous chimpanzee sequence 57 and human 2xHAR.142 gained developmental enhancer activity in the neocortex compared with the chimpanzee and mouse orthologs 58. Similarly, Capra and collaborators tested 29 HARs with epigenetic signatures of active developmental enhancers in transgenic mouse reporter assays. They found 24 HARs that act as developmental enhancers at E11.5 in specific embrionic tissues, and five of these show expression differences between the human and chimpanzee orthologous sequences 59. Thus, the human‐specific mutations in eight HARs had been shown to alter developmental enhancer function prior to the work of Boyd and colleagues. What was novel about their study was the discovery that mutations that occurred during human evolution can also change molecular and organismal phenotypes downstream of changes in enhancer activity.

## Linking human‐specific genetic changes to unique cognitive traits is a long and twisted road

Comparative studies of human and chimpanzee (or ancestral) enhancers are an important step towards linking human genetic changes to unique anatomical and molecular features of our brains. But even if these connections can be ellucidated, the challenge of associating human‐specific brain features with cognative abilities and traits will remain. In the case of HARE5, one specific question is how to link the observed differences in faster progenitor cell cycle in the developing brain and increased neocortical size to cognitive abilities, behavior, or language. One approach to this problem is to look directly for differences in phenotypes in humanized mice in which specific genes or non‐coding sequences have been engineered to match the human, rather than mouse, genome.

An example of this approach is the study of *FOXP2*, a developmental gene that likely underwent positive selection in the human lineage 60. *FOXP2* was dubbed a “language gene” after the discovery that a point mutation in the coding region led to aphasia in an English family 61, 62, 63, although this particular mutation was not one of the two carrying signatures of selection. To explore the functions of the positively selected amino acids, Enard and co‐workers replaced the endogenous mouse gene with a version carrying these two human‐specific changes 64. The resulting humanized mice where phenotipically characterized. Although they are overall healthy, they show some differences in qualitatively different ultrasonic vocalizations, decreased exploratory behavior and decreased dopamine concentrations in the brain, suggesting that the humanized Foxp2 allele affects basal ganglia 64. The major advance of this body of work is that it established a link between human‐specific mutations and several complex organismal phenotypes. What has not yet been done is to show the molecular pathways or developmental processes through which the genetic differences were expressed. This knowledge would advance our understanding of the mechanisms through which human evolution occurred. The study of Boyd and co‐workers is a first step toward filling in this black box between genotype and phenotype for a different human‐specific locus, though the authors have not gone as far as demonstrating effects on downstream phenotypes.

## How to link genotype to phenotype?

To fully understand differences between humans and chimpanzees we need to understand how genetic differences impact on phenotype. The studies of Enard et al. and Boyd et al. represent major advances in our understanding of the possible phenotypic effects of human‐specific genetic changes in genes and regulatory regions on neurodevelopmental programs. But we still have a long way to go to fully characterize the functions of these human‐specific genome sequences and how that function was changed during human evolution. Studies designed to link specific genetic differences to uniquely human traits involve many steps. A possible path starts by mapping human‐specific genetic differences and then analyzing the evolutionary forces underlying their appearance. Then it is necessary to assess function (particularly with non‐coding regions) and study anatomical and cellular differences between animals or cells with human versus chimpanzee versions. Finally, the most challenging step is to link molecular differences to human phenotypes.

Our progress on each of these steps is limited in various ways. One major issue is our dependence on model organisms to assess human genotype‐phenotype connections. Working with mice to model human brain evolution allows us to better understand how a particular change in a protein or regulatory region can impact on some aspects of molecular and cellular mechanisms of development leading to morphological differences. Rodents are not as distantly related to primates as commonly believed, as we belong to sister phylogenetic orders that group together in the super‐order Euarchontaglires, which evolved around 85–90 million of years ago 65. Humans and mice share many anatomical similarities that together with other characteristics, such as genetic tools and relatively low cost and easy husbandry, have made mouse the top model organism to study human diseases 66, 67. However, studying human biology in rodents has important limitations. It is clear that mice have differences in their brain anatomy and function that make them an imperfect system to study primate neurobiology. Similarly, they are not ideal for assessing human‐specific morphologies and behaviors. In addition, it is difficult to assess the function of a human regulatory sequence in a mouse, because of the myriad differences between human and mouse in other parts of the genome, including changes in transcription factors that bind to the sequence or changes in other regulatory regions. Hence, the function of a human sequence in the mouse genome cannot truly recapitulate its function in the native context of the human genome.

While it is not easy to imagine a solution for genetic studies at the organismal level there are some technological advances that are advancing this field. Induced pluripotent stem cells (iPSCs) 68 and techniques allowing their in vitro differentiation into various cell lines and tissues (reviewed in 69, 70) make it feasible to do genetics and study downstream cellular and molecular phenotypes in human and chimpanzee cells 71, 72, 3D cultures, and organoids 73, 74, 75, 76, 77. These systems have great potential to allow researchers directly to compare neurodevelopment in the presence and absence of DNA changes that occurred during human evolution. Another challenge is the shear number of HARs and HAR mutations to test for phenotypes. The field has identified a handful enhancers that gained or lost function in humans compared with the chimpanzee sequence. But there are still more than 2,500 HARs that need to be functionally characterized if we want to assess the impact of accelerated evolution in non‐coding regions on the evolution of our species. Massively parallel reporter assays 78, 79, which can be performed in iPSC‐derived cell lines, are one promising high‐throughput approach to screen large numbers of candidate enhancers and enhancer mutations.

## Conclusions and outlook

It is clear that no one technique or approach will shed light on the key genetic changes necessary to build a human. We hypothesize that conclusions about the particular developmental pathways and programs that were modified during the evolution of *Homo sapiens* will only be discovered through the use of a combination of approaches, including transgenic and mutant model organisms, comparative studies on iPSC‐derived cells and organoids from humans and non‐human primates, and human population genetics studies. Mechanistically to understand the evolution of humans will have a tremendous impact on philosophical, religious, and cultural matters. It will also have a fundamental affect on biomedical science, because understanding the molecular and cellular pathways that make us different will provide an unprecedented possibility to also explore whether these modified pathways are responsible for human susceptibility to particular diseases.
